# The Current Landscape of NKT Cell Immunotherapy and the Hills Ahead

**DOI:** 10.3390/cancers13205174

**Published:** 2021-10-15

**Authors:** Adam Nelson, Jordan D. Lukacs, Brent Johnston

**Affiliations:** 1 Department of Microbiology and Immunology, Dalhousie University, Halifax, NS B3H 4R2, Canada; adam.nelsonn@dal.ca (A.N.); JLukacs@dal.ca (J.D.L.); 2 Beatrice Hunter Cancer Research Institute, Halifax, NS B3H 4R2, Canada; 3 Department of Pediatrics, Dalhousie University, Halifax, NS B3H 4R2, Canada; 4 Department of Pathology, Dalhousie University, Halifax, NS B3H 4R2, Canada

**Keywords:** natural killer T cells, iNKT cells, type II NKT cells, cancer, immunotherapy, α-galactosylceramide, CAR-NKT cells, adoptive transfers, checkpoint inhibitors, cancer vaccines

## Abstract

**Simple Summary:**

Natural killer T (NKT) cells are a subset of lipid-reactive T cells that enhance anti-tumor immunity. While preclinical studies have shown NKT cell immunotherapy to be safe and effective, clinical studies lack predictable therapeutic efficacy and no approved treatments exist. In this review, we outline the current strategies, challenges, and outlook for NKT cell immunotherapy.

**Abstract:**

NKT cells are a specialized subset of lipid-reactive T lymphocytes that play direct and indirect roles in immunosurveillance and anti-tumor immunity. Preclinical studies have shown that NKT cell activation via delivery of exogenous glycolipids elicits a significant anti-tumor immune response. Furthermore, infiltration of NKT cells is associated with a good prognosis in several cancers. In this review, we aim to summarize the role of NKT cells in cancer as well as the current strategies and status of NKT cell immunotherapy. This review also examines challenges and future directions for improving the therapy.

## 1. Introduction

Cancer is a leading cause of death globally [1]. Current cancer treatments including, chemotherapy and radiation, are not always effective, and have severe adverse effects, leading to many dose-limiting toxicities [2,3]. Furthermore, genetic mutations, epigenetic changes, and increased drug efflux lead to chemotherapy resistance, with 90% of cancer-related deaths due to drug resistance and metastasis [4,5,6]. Therefore, new therapies that can safely and effectively treat cancer are needed. One emerging treatment option is immunotherapy. Immunotherapy using checkpoint inhibitors, adoptive cell transfers, and chimeric antigen receptor (CAR) T cells have recently seen great preclinical and clinical success [7,8,9]. Natural killer T (NKT) cell-based immunotherapy has also seen great preclinical success [10,11,12]. However, clinical translation has been challenging. In this review, we describe the strategies used in preclinical and clinical NKT immunotherapy studies, as well as challenges and future directions for NKT cell-based immunotherapies.

## 2. NKT Cells

NKT cells are a subset of lipid and glycolipid-reactive T lymphocytes that co-express markers associated with NK cells (NKp46, NK1.1) [13,14]. NKT cells play an important role in tumor immunosurveillance and anti-tumor immunity [15,16,17]. Unlike traditional T cells that recognize peptide antigens in the context of MHC I or II, NKT cells recognize endogenous and exogenous glycolipids presented via the MHC I-like molecule CD1d [18]. NKT cells can be separated into two major subsets: type I and type II NKT cells based on TCR rearrangements and glycolipid reactivity [19]. Type I or invariant NKT (iNKT) cells express an invariant TCRα chain rearrangement paired with a restricted repertoire of TCRβ chains; Vα14Jα18 (TRAV11-TRAJ18) paired with Vβ7 (TRB29), Vβ8.2 (TRB13-2), or Vβ2 (TRBV1) in mice and Vα24Jα18 (TRAV10-TRAJ18) paired with Vβ11 (TRBV25-1) in humans [20]. Upon recognition of glycolipid antigens, iNKT cells rapidly secrete immunomodulatory cytokines, including IFNγ, TNF, and IL-4, to influence downstream immune activity [21,22]. Human iNKT cells can be CD4^+^, CD8^+^ double-negative (CD4^−^ CD8^−^), whereas NKT cells in mice are either CD4^+^ or CD4^−^CD8^−^ [23,24,25]. This is due to mouse iNKT cells expressing the transcription factor Th-POK, which is necessary for proper iNKT development, but silences CD8 expression [26]. Furthermore, iNKT cells can be broken down into NKT1, NKT2, and NKT17 subsets, mirroring the T_h_1, T_h_2, and T_h_17 T cell subsets [21,25]. Type II NKT cells have a more diverse repertoire of Vα rearrangements (including TRAV7, TRAV9, and TRAV12) [27,28] that facilitate recognition of self-lipids such as sulfatide or lysophosphatidylcholine [29,30]. Multiple studies have shown that activation of type II NKT cells via administration of sulfatides increases tumor growth and enhances metastasis, suggesting a pro-tumor role, whereas iNKT cells exhibit robust anti-tumor activity [31,32].

## 3. iNKT Cells in Cancer

**Figure 1:**
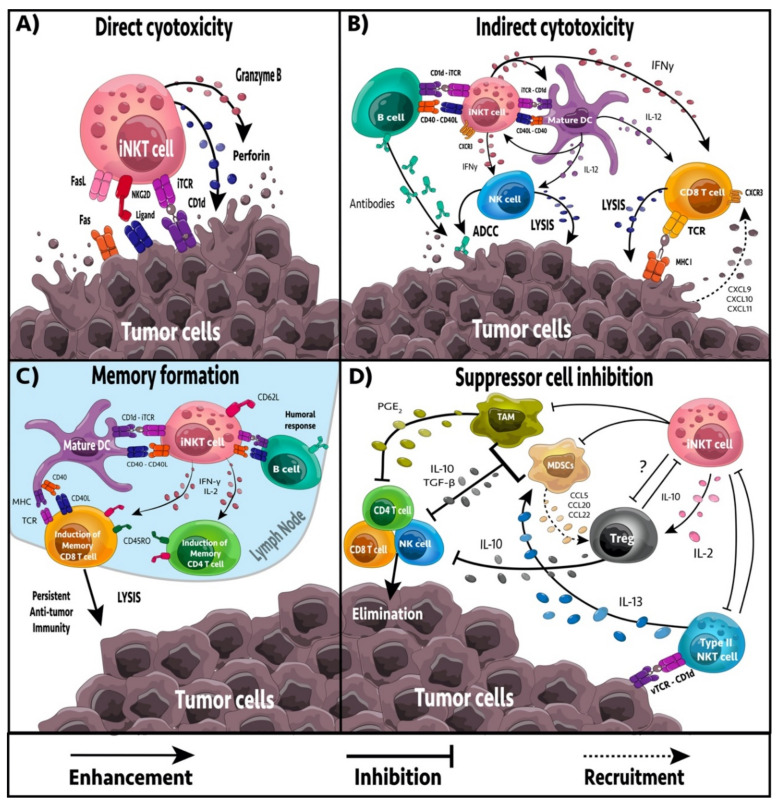
The complex network between iNKT cells and other immune cells in the tumor microenvironment. (**A**) iNKT cells activated by antigenic, or cytokine stimulation can promote anti-tumor immunity by directly eliminating tumor cells through various cell-to-cell interactions (e.g., invariant TCR → antigen-loaded CD1d, NKG2D → ligand) that induce the release of cytotoxic granules (e.g., granzyme B, perforin) or apoptotic signaling (e.g., FasL → Fas). (**B**) iNKT cells and other effector lymphocytes may be recruited to the TME via chemokines (e.g., CXCL9, CXCL10, CXCL11, CXCL16) released via immunogenic cell death from tumor cell lysis, or from other tumor-infiltrating immune cells. iNKT cells activated by antigen-presenting cells (APCs) such as dendritic cells (DCs) or B cells can indirectly promote anti-tumor immunity. Upon cognate TCR-CD1d interactions, iNKT cells upregulate CD40L that induces APC maturation and release cytokines that mediate activation of other effector lymphocytes (NK cells and CD8 T cells). (**C**) iNKT cells may also induce long-lasting anti-tumor immunity by promoting the formation of effector memory CD4 and CD8 T cells that are tumor specific. iNKT cell-mediated activation of DCs or B cells promotes the presentation of tumor antigens to CD4 and CD8 T cells. iNKT cell-derived cytokines also promote expansion and differentiation of effector and memory T cells, further promoting persistent anti-tumor immunity. (**D**) iNKT cells also interact with suppressor cells in the TME, including T regulatory (Treg) cells, tumor-associated macrophages (TAMs), myeloid-derived suppressor cells (MDSCs), and type II NKT cells. Tregs and type II NKT cells cooperate to cross-regulate iNKT cells in the tumor and inhibit their anti-tumor functions. Tregs, MDSCs, and TAMs can produce IL-10 to promote tolerance and inhibit the effector functions of neighboring CD4 T, CD8 T, and NK cells. MDSCs and TAMs may also inhibit effector lymphocytes via the production of TGF-β and PGE_2_, whereas PGE_2_ induces Tregs. Type II NKT cells may recruit and activate MDSCs via IL-13, which consequently enhances their TGF-ß production. MDSCs may recruit Tregs into the TME via chemokines such as CCL5, CCL20, or CCL22. iNKT cells oppose the suppressive functions of MDSCs and TAMs by polarizing macrophages towards a favorable M1 phenotype, inhibiting MDSC suppressive functions, or by lysing MDSCs/TAMs.

It is well established that iNKT cells have a direct role in anti-tumor immunity and immunosurveillance. Treatment with the chemical carcinogen, methylcholanthrene increased the incidence of tumors in Jα18^−/−^ iNKT cell-deficient mice [33]. Transgenic adenocarcinoma of the mouse prostate (TRAMP) mice deficient in iNKT cells exhibited increased tumor development and metastasis, leading to reduced survival compared to wild-type TRAMP mice [34]. Furthermore, Jα18^−/−^ p53^−/−^ double knockout mice succumb to tumors faster than p53^−/−^ knockout mice, further demonstrating NKT cell role in cancer immunosurveillance [11]. In humans, cancer patients often present with reduced numbers of iNKT cells and/or impaired iNKT cell function [35,36,37,38]. In contrast, iNKT cell infiltration into tumors is associated with a good prognosis in chronic lymphocytic leukemia, neuroblastomas, colorectal carcinoma, and pancreatic adenocarcinoma [39,40,41,42,43]. iNKT cell immunosurveillance is likely mediated by inflammatory cytokines or recognition of tumor-associated glycolipids or stress-induced glycolipid antigens presented by CD1d positive tumor cells, or by antigen-presenting cells (APCs) [44,45,46,47]. CD1d positive tumors are more susceptible to iNKT cell-mediated lysis compared to CD1d negative tumors, and cancer cells often avoid detection by downregulating CD1d, demonstrating an important role for CD1d-TCR interactions in immunosurveillance [48,49,50]. Therapeutic activation of iNKT cells via exogenous glycolipids, such as α-galactosylceramide (α-GalCer), increases anti-tumor immunity and provides protection from tumor progression [51,52,53]. Activated iNKT cells can provide anti-tumor immunity by four mechanisms: direct tumor lysis, recruitment and activation of cytotoxic innate and adaptive immune cells, inhibition of suppressive cells in the tumor microenvironment (TME), and promotion of tumor-targeted immune memory (Figure 1).

## 4. iNKT Cell-Mediated Anti-Tumor Effector Responses

iNKT cells can target tumor cells both directly and indirectly. iNKT cells have the capacity to mediate direct cytolytic activity against CD1d positive tumor cells via perforin, granzyme B, and TNF-related apoptosis-inducing ligand (TRAIL) pathways [54]. In vitro and in vivo studies have linked iNKT cell-mediated cytotoxicity with increased expression of CD1d on tumor cell surfaces, leading to enhanced tumor cell lysis and reduced metastasis rate, whereas downregulation of CD1d is associated with reduced iNKT cell recognition, tumor escape and increased malignancy [15,32,50,55]. However, tumors with low CD1d levels are not completely exempt from iNKT cell killing. A subset of human iNKT cells expressing the stress ligand-receptor NKG2D can engage in an NK-cell-like cytolytic response against cells expressing NKG2D ligands [56]. NKG2D^+^ iNKT cells could initiate killing in the absence of TCR recognition of CD1d, demonstrating an important role in targeting tumors that lack CD1d expression [56].

In the absence of CD1d expression on tumor cells, iNKT cells may become activated by CD1d^+^ APCs including dendritic cells (DCs), B cells, myeloid-derived suppressor cells (MDSCs), and tumor-associated macrophages (TAMs) [51,57,58,59,60]. The iNKT cell response depends largely on the APC presenting the glycolipid. Glycolipid presentation by DCs leads to the strongest stimulation due to the presence of co-stimulatory signals [61]. Presentation of glycolipid by B cells, in comparison, leads to a weak iNKT response and a more anergic phenotype [60]. Furthermore, B cell presentation of α-GalCer can skew iNKT cell cytokine responses to a T_h_2 phenotype [62], whereas DCs skew towards T_h_1, creating a more desirable anti-tumor immune response [61]. Upon activation, iNKT cells can secrete large amounts of pro-inflammatory cytokines including IL-2, IL-4, IL-17, IFNγ, and TNF [21,22,63], which affect a broad spectrum of immune cells, including DCs, macrophages, neutrophils, NK cells, and T and B cells. The ability to activate both NK cells and CD8^+^ T cells is critical as it allows for the targeting of both MHC negative and MHC positive tumors, overcoming MHC downregulation as an immune escape mechanism [64]. Activated iNKT cells actively recruit and induce the maturation of DCs through CD40/CD40L and CD1d/TCR interactions [65]. DCs residing in the TME are primarily immature and inept at activating T cells [66,67]. Interactions between glycolipid-presenting DCs and iNKT cells upregulate costimulatory molecules on DCs, such as CD40, CD80, and CD86, and induces the production of IL-12 and CXCL16 [51,68,69]. CXCL16 enhances IFNγ production by NKT cells, while IL-12 enhances IFNγ secretion by iNKT cells, NK cells, T_h_1 CD4^+^ and CD8^+^ T cells, providing a positive feedback loop to support a robust anti-tumor immune response [70]. iNKT cell recruitment of B cells into the TME can enhance T cell responses by in part serving as APCs and presenting tumor antigens, as well as, polarizing lymphocytes towards T_h_1, T_h_2 or other functional states by secreting IFNγ and IL-4 [71,72,73].

## 5. Regulation of Immune Suppression

TAMs are immunosuppressive immune cells found in high frequency in the TME. TAMs contribute to tumor progression and suppression of NK, iNKT, and T cell responses [74]. In neuroblastomas, iNKT cells can co-localize and lyse TAMs via a CD1d dependent mechanism [58,75]. Furthermore, iNKT cells can reprogram M2 polarized TAMs to inflammatory M1 macrophages, reducing TAM mediated immunosuppression [76,77]. MDSCs are populations of undifferentiated and immunosuppressive myeloid cells that accumulate in cancer patients [78]. MDSCs induce anergy in NK and T cells, contributing to immune suppression and tumor growth [79]. Furthermore, they can remodel the TME, increasing the rate of metastasis [80]. In an influenza A infection model, iNKT cells could inhibit arginase-1 and NOS2 mediated suppressive activity of MDSCs in a CD1d and CD40 dependent manner [59]. Activation of iNKT cells by α-GalCer-loaded DCs also leads to reduced frequency and inhibitory function of MDSCs in a 4T1 breast cancer model [52]. Furthermore, NKT cells can stimulate the conversion of α-GalCer-loaded MDSCs into mature APCs capable of eliciting NK and T cell immune responses [81]. Type II NKT cells are found in high frequency in the TME and are immunosuppressive, increasing tumor progression and metastasis [31,82]. Activation of iNKT cells decreases type II NKT cell number and function in a cross-regulation mechanism, reminiscent of the T_h_1-T_h_2 axis [31,32,82,83]. Similarly, stimulation of type II NKT cells by sulfatides decreases iNKT cell proliferation and cytokine secretion, leading to increased tumor progression [84]. As sulfatides and other immunosuppressive glycolipids accumulate in some cancers [85,86,87,88,89], this could impact immunosurveillance by iNKT cells.

## 6. Formation of Tumor Immune Memory

Generation of immune memory is critical for long-lasting anti-tumor immune responses and preventing tumor relapse [90]. Activation by tumor antigens or cross-reacting foreign antigens induces naïve T cells to undergo expansion and differentiation to acquire the appropriate effector functions to target cancer cells. Co-delivering antigens and adjuvants to DCs is a well-known strategy for inducing optimal T and B cell immune responses [91]. CD8α^+^ DCs excel in MHC I cross-presentation and iNKT cells have been shown to directly license CD8α^+^ DCs for cross-priming even in the absence of CD4^+^ T cells [92]. A previous study examined the targeted delivery of α-GalCer to CD8α^+^ DCs via anti-DEC205 decorated nanoparticles in an attempt to improve iNKT cell-based anti-tumor responses in a murine B16-F10 melanoma model [92]. Delivery of α-GalCer to CD8α^+^ DCs mediated activation of iNKT cells and downstream activation of NK and γδ T cells via cross-talk-induced IL-12. Importantly, α-GalCer-loaded DCs amplified the primary and memory CD8^+^ T cell responses in an iNKT cell-dependent manner, leading to persistent anti-tumor immunity [92,93]. NKT cell activation has similarly been shown to boost CD8^+^ T cell-mediated anti-tumor immunity in other tumor models [52].

While most iNKT cells lack CD62L [94,95], a recent study has demonstrated that CD62L^+^ iNKT cells were increased after glycolipid stimulation and persisted longer than CD62L^neg^ iNKT cells [96]. CD62L^neg^ iNKT cells acquire an exhausted phenotype (PD-1, TIM-3, reduced cytokine production) and rapidly undergo apoptosis whereas CD62L^+^ iNKT cells continued to proliferate and produce large amounts of cytokines [96]. Using a murine model of B cell lymphoma, these authors found that CD62L^+^ iNKT cells transduced with a CD19-specific CAR achieved sustained tumor regression and enhanced survival [96]. In contrast, mice that received CD62L^-^ iNKT CD19 CAR cells succumbed to tumor progression. 

## 7. iNKT Cell Immunotherapy

Due to the important role of iNKT cells in immunosurveillance and anti-tumor immunity, iNKT cell-based immunotherapy has been a great area of interest. The overall efficacy of iNKT cell immunotherapy is largely dependent on the number and function of tumor-infiltrating iNKT cells and APCs, and often correlates with the level of CD1d expression on tumor cells. Multiple strategies have been used to target cancer using iNKT cell-based immunotherapeutic approaches (Table 1). While many preclinical studies have shown iNKT cell immunotherapy strategies to be safe and effective, not all strategies are clinically effective. Therefore, it is important to consider the preclinical and clinical data for each strategy moving forward.

### 7.1. Free α-GalCer Administration

In preclinical trials, free α-GalCer can be given prophylactically, simultaneously, or after tumor inoculation. While prophylactic and simultaneous treatments are important in the context of cancer vaccines, treatment after tumor establishment is more relevant to the application of immunotherapy. Upon free α-GalCer injection, iNKT cells rapidly proliferate and produce TNF, IL-2, IL-4, and IL-13, followed by a later IFNγ response [63], with multiple injections skewing towards T_h_2 response [138]. Furthermore, multiple injections of free α-GalCer induce anergy of NKT cells leading to increased PD-1 expression and reduced cytokine expression and cell numbers [138,139]. While free α-GalCer administration post tumor inoculation has shown some anti-tumor effect in multiple murine models, the anti-tumor response is limited [97,98,99,100]. Therefore, free α-GalCer is often given in combination with cytokines, or other immunotherapies to increase the efficacy of the treatment [117,122]. In a phase I clinical trial, administration of free α-GalCer was well tolerated, having no dose-limiting toxicity; however, it failed to yield a clinical response with only 7 of 24 patients achieving stable disease [140]. The low efficacy can be attributed to low iNKT cell numbers at baseline, as only patients with high numbers of iNKT cells exhibited NK cell cytotoxicity and stable disease, Similarly, anergy was likely to limit efficacy as functional responses decreased with subsequent injections of α-GalCer [140].

### 7.2. Adoptive Transfer of DCs Presenting α-GalCer

DCs loaded with α-GalCer have been used to overcome the low immunogenicity and anergy induction of free α-GalCer. Loading of α-GalCer on DCs leads to a stronger activation of iNKT cells and less induction of anergy due to the co-signaling of CD40 and IL-12 [44,51]. In multiple tumor models, therapeutic administration of α-GalCer-loaded-DCs reduced tumor growth and metastasis, resulting in prolonged survival compared to free glycolipid administration [51,52,102]. α-GalCer-loaded-DCs increased iNKT expansion, activation, and IFNγ production [51,52]. Furthermore, enhanced activation of iNKT cells leads to increased activation and IFNγ production from NK and CD8 T cells [44]. Multiple clinical studies have used autologous APCs pulsed with α-GalCer. Both immature and mature DCs have been used in clinical trials, with mature DCs being more effective, due to their increased ability to activate iNKT cells [44,103,133,141]. In clinical trials of myeloma and head and neck cancer, mature α-GalCer-loaded-APCs were well tolerated with no serious adverse events [103,133]. Treatment increased IFNγ production and expansion of iNKT cells, leading to stable disease in many patients and increased median survival times. In a phase I and phase I/II clinical trial of non-small cell lung cancer, treatment with α-GalCer-loaded-APCs was well tolerated, resulting in only grade I and II adverse events [104,142]. Patients who responded well to treatment exhibited increased IFNγ production and expansion of iNKT cells. The increased immune response led to a median survival time of 31.9 months, compared with the median survival time of all patients being 18.6 months [142]. A follow-up study by Toyoda et al. [105], examined overall survival time two years post-treatment and found a median overall survival of 21.9 months. Both studies demonstrate that α-GalCer-loaded-DCs can be an effective treatment option for NSCLC. Although α-GalCer-loaded-DCs exhibited a good immune response, there are still factors that limit efficacy. Treatment responses in these studies were dependent on the baseline number and function of iNKT cells in the patients.

### 7.3. Adoptive Transfer of Activated iNKT Cells

Cancer patients often have low numbers and/or reduced function of iNKT cells, limiting the efficacy of α-GalCer treatments [35,36,37,38]. To overcome this limitation, iNKT cells have been isolated from patient-derived PBMCs and expanded ex vivo before adoptive transfer back into the patient. In preclinical models, adoptive transfer of activated iNKT cells increased iNKT cell cytotoxicity, tumor regression, and overall survival in a metastasis model of melanoma [106]. In contrast, NKT cell transfer alone or in combination with free α-GalCer therapy or glycolipid-loaded DCs did not improve outcomes in a model of metastatic 4T1 breast cancer metastasis [52]. Clinical trials using iNKT cell adoptive transfer in non-small lung cancer and advanced melanoma reported no serious adverse events [17,107]. Patients exhibited increases in circulating iNKT cell numbers and IFNγ production, but few patients showed any reduction in tumor progression [17,107], indicating the treatment was not effective. Combining the adoptive transfer of expanded iNKT cells with α-GalCer-loaded-DCs increased the efficacy of the adoptive NKT cell transfer [116]. Patients receiving the combined therapy exhibited further increases in circulating iNKT cell numbers and IFNγ production compared to iNKT adoptive transfer alone, with some patients achieving a partial response [116].

### 7.4. iNKT Cell Ligands as Cancer Vaccine Adjuvants

The role of iNKT cells in recognizing conserved lipid antigens presented via CD1d, and their ability to orchestrate the anti-tumor immune response through cytokine signaling make them attractive targets for cancer vaccine development. Ligands for iNKT cells, such as α-GalCer, can be co-administered with tumor antigens and function as an adjuvant, increasing the adaptive immune response towards the tumor. When given before or after tumor inoculation, α-GalCer co-formulated with tumor antigens increased overall survival in mice, a protective effect that was largely dependent on enhanced CD4 and CD8 T cell responses [143,144,145]. Intranasal vaccination with α-GalCer combined with OVA-induced both humoral and cellular immune responses, resulting in increased cytokine secretion, CTL responses, and IgG production [146]. Importantly, vaccination of CD1d^−/−^ mice does not induce anti-tumor responses, demonstrating that the presentation of α-GalCer to iNKT cells is essential [143,146]. The anti-tumor immune response can be further enhanced by using α-GalCer-loaded-DCs or CD1d expressing tumor cells loaded with α-GalCer [101,147]. Vaccination with irradiated α-GalCer-loaded autologous tumor cells prevented the formation of lymphoma tumor deposits [101]. Furthermore, this protection was lost in Jα18^−/−^ iNKT cell-deficient mice, demonstrating that iNKT cells were essential in the protection. Currently, there are no registered clinical trials using iNKT cell ligands in cancer vaccines.

### 7.5. CD1d-Antibody Fusion Proteins

CD1d positive tumors or more susceptible to iNKT cell-mediated lysis, and downregulation of CD1d is a common evasion tactic to avoid detection [48,49,50]. Therefore, to increase the iNKT cell targeting of tumors, CD1d-antibody fusion proteins that direct iNKT cell lysis towards the tumor have been examined. The N-terminus of CD1d is fused to β2-microglobulin to create a soluble CD1d (sCD1d) [114]. A single-chain variable fragment (scFv) against the desired tumor marker is bound to the sCD1d via a linker. The CD1d-antibody fusion protein is then loaded with α-GalCer before administration [114]. Currently, CD1d-antibody fusion proteins targeted against human epidermal growth factor receptor 2 (HER2), CEA, and CD19 have been developed [113,114,115]. In a B16 melanoma model expressing HER2, treatment with a CD1d-antibody fusion protein targeting HER2 increased iNKT cell inflammatory cytokine production and targeted lysis of tumor cells, decreasing metastasis formation [114]. Furthermore, iNKT cells enhanced activation of DCs, NK cells and CD8 T cells, increasing overall immune recruitment and targeting of the tumor [114]. Similar results were observed using CD1d-antibody fusion proteins targeting CEA and CD19 [113,115,148]. Importantly, the directed lysis of iNKT cells by CD1d-antibody fusion proteins was specific, and caused limited off-target effects, demonstrating a good safety profile [113].

### 7.6. CAR-NKT Cells

**Figure 2:**
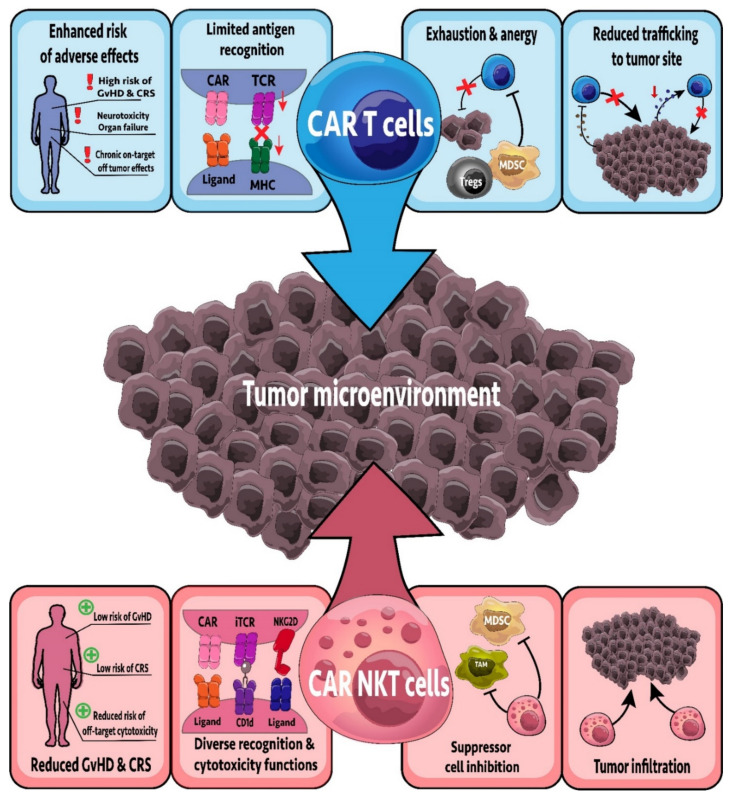
CAR-NKT cells versus CAR-T cells; how do they measure up? CAR-NKT cells have several advantages over their CAR-T cell counterparts. For example, CAR-T cells can induce graft versus host disease (GvHD), cytokine release storms (CRS), neurotoxicity, and on-target off-tumor side effects in the recipient. This is often due to a limited repertoire of antigen recognition receptors (e.g., TCR, CAR receptors) and higher secretion of cytokines such as IL-6 causing CRS. CAR-NKT cells instead display a much-improved safety profile with minimal side effects due to their distinct cytokine profile (failing to induce CRS) and a broad spectrum of antigen recognition receptors in addition to their CAR receptor. CAR-T cells are also challenged with the inability to counteract the suppressive nature of the tumor microenvironment, and often succumb to exhaustion or anergy. CAR-NKT cells can overcome immunosuppression by targeting and inhibiting myeloid-derived suppressor cells (MDSCs), tumor-associated macrophages (TAMs) via cytotoxic effects or macrophage polarization, respectively. CAR-NKT cells may also be stimulated via glycolipids such as α-GalCer and can infiltrate the solid tumor, unlike CAR-T cells which frequently exhibit impaired trafficking to the tumor site.

CAR-T cell therapies have yielded significant clinical success in targeting B cell malignancies. CAR-T cells expressed a chimeric antigen receptor composed of the transmembrane domain and endodomain of CD3ζ linked to an scFv of a monoclonal antibody that recognizes a specific antigen. CAR-T cells engineered to target CD19 can provide curative outcomes for patients with B cell acute lymphoblastic leukemia, with many CAR-T cells targeting different antigens currently in clinical trials [149]. However, there are many reasons why expressing CARs in innate cells may be better than traditional CAR-T cells, including decreased cytokine release storms (CRS) and graft vs. host disease (GvHD) (Figure 2) [150]. iNKT cells have been engineered to express CARs targeting glycolipid and protein antigens. Furthermore, CAR-NKT cells co-express the invariant TCR in addition to the CAR, maintaining their reactivity to glycolipid antigens. In contrast, the endogenous TCR in CAR-T cells is polyclonal and most CAR-T cells would only react via their CAR [111]. CAR-NKT cells targeting CSPG4, GD2, and CD19 have been developed and examined preclinically, with CAR-NKT cells targeting GD2 and CD19 also being tested in clinical trials [96,108,112,151].

GD2 is a glycosphingolipid highly expressed in melanoma, neuroblastoma, and some patients with other solid tumors [152,153,154]. The role of GD2 in cancer is not well understood, but recent work has implicated functions in tumorigenesis, cancer metabolism, and metastasis [153,155]. Anti-GD2 CAR-NKT cells localized to the tumor and exhibited a strong cytotoxic effect in vivo, decreasing tumor burden and increasing overall survival [109]. Furthermore, the cytotoxicity and survival benefit could be improved by engineering the CAR-NKT cell to co-express IL-15 [109], an important survival cytokine for iNKT cells [156,157]. Importantly, unlike anti-GD2 CAR-T cells, anti-GD2 CAR-NKT cells did not cause GvHD, demonstrating increased safety [109,158]. Due to the preclinical success in neuroblastoma, a phase I clinical trial examining anti-GD2 CAR-NKT cells in refractory neuroblastoma has commenced (NCT03294954). Initial results show that treatment is safe in the ten patients enrolled, with one complete response, one partial response, and three patients with stable disease [110]. While GD2 is also highly expressed in melanoma, anti-GD2 CAR-NKT cells have not been tested in a melanoma model. However, CAR-T cells targeting GD2 in melanoma have realized good results [158], warranting the examination of anti-GD2 CAR-NKT cells in melanoma.

Anti-CD19 CAR-T cells can provide curative outcomes for patients with B cell malignancies [149]. Therefore, anti-CD19 CAR-NKT cells have also been examined in B cell malignancies. In a B cell lymphoma model, CD62L^+^ CD19 specific CAR-NKT cells had persistent anti-tumor effects leading to increase survival [96]. Furthermore, co-stimulation with α-GalCer further enhanced the anti-tumor function of anti-CD19 CAR-NKT cells, demonstrating that co-stimulation through CD1d and the CAR TCR can provide increased therapeutic benefit [111]. Importantly, this treatment strategy was more effective than anti-CD19 CAR-T cell treatment, and effectively eliminated secondary lymphoma, indicating that anti-CD19 CAR-NKT cells may provide better therapeutic outcomes than traditional CAR-T cells [111]. Currently, a phase I clinical trial is underway, examining anti-CD19 CAR-NKT cells in relapsed and refractory B cell malignancies (NCT03774654). While only initial results have been released, Kuur Therapeutics have indicated that of the two patients in the phase I trial, one exhibited a complete response and the other a partial response, with both patients lacking any signs of CRS or GvHD [159]. Importantly, the CAR-NKT cells used in this study were allogenic, demonstrating the potential for an “off the shelf” therapy. One potential limitation is that allogenic NKT cells likely express MHC molecules that would be recognized by the recipient as foreign, leading to rejection of the transferred NKT cells. However, strategies to knockout or knockdown MHC on engineered CAR cells could mitigate this [150].

### 7.7. Combination Therapies

One way to improve upon existing therapies is to combine them with other therapeutics that generate synergistic responses. Indeed, combinations of different chemotherapeutics and immunotherapies, such as checkpoint inhibitors, are being explored for treatment for cancer patients [160,161,162,163,164]. Therefore, a combination approach may also increase clinical outcomes for iNKT cell immunotherapies. In preclinical experiments, iNKT cell immunotherapy has been combined with chemotherapy, oncolytic viruses, and other immunotherapies (Table 1). While there are many preclinical approaches under investigation, clinical trials examining combination therapies that incorporate iNKT cell immunotherapy have been limited.

#### 7.7.1. Combination with Chemotherapy

While combining immunotherapy with chemotherapy, the choice of chemotherapeutic is critical. Chemotherapies target proliferating cells, therefore immune cells can also be targeted, making some chemotherapy agents immunosuppressive. Indeed, some chemotherapies, such as taxanes and continuous doses of cyclophosphamide, cause myelosuppression and leukocytopenia which can limit the efficacy of subsequent immunotherapy [165,166]. However, some chemotherapies have shown immunostimulatory effects, turning cold tumors hot and increasing the efficacy of subsequent immunotherapy [167,168]. Many chemotherapies lead to the release of damage-associated molecular patterns and chemokines, resulting in immunogenic cell death (ICD), a type of cell death that recruits and activates innate immune cells and primes adaptive responses against the tumor [169]. In vitro, pretreating cell lines with cisplatin, doxorubicin, methotrexate, or etoposide sensitized tumor cells to iNKT cell cytotoxicity via TRAIL and FasL [170,171]. Furthermore, pretreatment with gemcitabine or cyclophosphamide, chemotherapies that induce ICD, increased iNKT cell immunotherapy efficacy and survival in an in vivo 4T1 breast cancer model, although tumor-targeted NK and CD8 T cell responses were not higher than with NKT cell activation alone [172]. While chemotherapies that induce ICD increase subsequent iNKT cell immunotherapy, chemotherapies that do not induce ICD, such as cisplatin and 5-Florouracil (5-FU), can still increase subsequent iNKT cell immunotherapy. While cisplatin and 5-FU do not increase ICD, they do increase immune activity in the tumor, possibly through the release of tumor antigens by dying cancer cells that are taken up and presented by DCs [131,132]. The presentation of tumor antigens to T cells increases immune activity in the tumor, making the tumor hot and increasing the efficacy of subsequent immunotherapy [173]. In a mesothelioma model, pretreatment of tumors with cisplatin increased gene expression of IFNγ and granzyme B, which could be further increased by subsequent α-GalCer administration, resulting in increased tumor regression [131]. In a colon cancer model, pretreatment of tumors with 5-FU increased NK cell cytotoxicity and coreceptor expression, increasing the efficacy of subsequent α-GalCer treatment [132]. Both studies demonstrate that chemotherapy does not need to increase ICD to increase the efficacy of iNKT cell immunotherapy.

Combination therapy with lenalidomide has generated the most success of any chemotherapy. Lenalidomide increases cyclin-dependent kinase inhibitors, decreasing cancer cell proliferation [174]. Lenalidomide treatment also decreases the expression of vascular endothelial growth factors, limiting tumor angiogenesis [175]. Furthermore, Lenalidomide increases T cell proliferation and NK cell function to enhance the anti-tumor immune response [176]. Pretreatment with lenalidomide increases iNKT cell expansion, activation, and IFNγ production both in vitro and ex vivo [57]. Furthermore, patients with myelodysplastic syndrome treated with lenalidomide exhibited increased iNKT cell cytokine production, demonstrating similar effects in vitro and in vivo [177]. To date, lenalidomide is the only chemotherapy to be combined with iNKT cell immunotherapy in clinical trials. In a phase I clinical trial, patients with asymptomatic myeloma were treated with α-GalCer-loaded-DCs in combination with lenalidomide and then continuous treatment with lenalidomide [133]. Treatment was well tolerated with only one patient exhibiting a grade 3 adverse event. Treatment led to increased activation of iNKT cells, NK cells, monocytes, and eosinophils, demonstrating strong innate immune activation [133]. Furthermore, NKG2D expression was significantly increased on NK cells, indicating increased NK cell cytotoxic potential [133]. Overall, the treatment led to decreased tumor-associated immunoglobulin in all but one patient, indicating that treatment decreased tumor burden [133]. The success of the combination therapy highlights the need for further clinical trials.

#### 7.7.2. Combination with Oncolytic Viruses

Oncolytic viruses selectively infect and lyse cancer cells by taking advantage of dysregulated signaling pathways important that normally limit virus infection and replication [178,179]. While oncolytic viruses can directly kill cancer cells by oncolysis, there is increasing evidence that oncolytic viruses also stimulate anti-tumor immunity [180,181]. Oncolytic virus infection increases immune cell activation and antigen presentation [135]. Furthermore, oncolytic viruses are potent inducers of ICD, therefore neoadjuvant treatment with oncolytic viruses may increase iNKT cell immunotherapy efficacy [182]. In models of ovarian and breast cancer, oncolytic reovirus and vesicular stomatitis virus (VSV) were able to increase the efficacy of iNKT cell immunotherapy by promoting enhanced survival [135]. However, only VSV was able to increase survival in the breast cancer model, demonstrating that the ability of specific oncolytic viruses to enhance iNKT cell immunotherapy efficacy could depend on the tumor. In this study, oncolytic VSV infected a broader range of cancer cells than reovirus. Another study demonstrated that vaccina virus expressing 15-ydroxyprostaglandin dehydrogenase, an enzyme that inactivates immunosuppressive prostaglandin E_2,_ can sensitize resistant tumors to iNKT cell adoptive transfer [134]. To date, no clinical trials have combined oncolytic viruses with iNKT cell immunotherapy. However, clinical trials have shown that oncolytic viruses can improve the effects of other immunotherapies [183]. Taken together, the preclinical success of combining oncolytic virotherapy with iNKT cell activation therapy suggests that further examination of these combination therapies in clinical trials is warranted.

#### 7.7.3. Combination with Immunotherapy

In several clinical trials of head and neck cancer, combination treatment of ex vivo activated iNKT cells and α-GalCer-loaded-DCs led to increased circulating iNKT cells and IFNγ production, leading to stable disease or tumor regression in patients [17,116]. However, the combination treatment did lead to more adverse advents when compared to monotherapy. One interesting option for combination therapy is to combine iNKT cell therapies with immune checkpoint inhibitors. Immune checkpoint antibodies that block inhibitory immune signaling receptors have achieved success in both preclinical and clinical settings [184,185,186]. For example, the inhibitory immune checkpoint receptors PD-1 and CTLA-4 are upregulated on activated leukocytes, limiting their activation and anti-tumor functions [187,188,189,190]. Activation of iNKT cells by α-GalCer leads to robust IFNγ release followed by increased expression of PD-1, resulting in anergy and inhibition of anti-tumor function [117,191]. Therefore, the combination of iNKT cell immunotherapy with checkpoint inhibitors blocking the PD-1/PD-L1 axis may increase therapeutic efficacy. Indeed, α-GalCer followed by anti-PD-1 or anti-PD-L1 prevented iNKT cell anergy increasing anti-tumor activity of the iNKT cells in preclinical models [117,118,119,191]. Furthermore, iNKT cell immunotherapy can overcome CD8^+^ T cell anergy in PD-1 resistant tumors [192]. Patients who respond to PD-1 therapy have increased peripheral blood iNKT cells [193], suggesting an important role for iNKT cells in the therapy response, with clinical trials examining the combination of PD-1/PDL-1 blockade and iNKT cell immunotherapy underway (NCT03897543). While combination therapy of iNKT cells with other checkpoint inhibitors such as anti-CTLA-4 is lacking, iNKT cells have been shown to be important in regulating the immune response in anti-CTLA-4, with mice lacking iNKT cells having reduced therapeutic benefit [194]. Taken together, clinical trials examining iNKT cell immunotherapy with checkpoint inhibitors are warranted.

Another combination strategy is combining iNKT cell immunotherapy with an immunostimulatory agent, such as monoclonal antibodies or cytokines. Monoclonal antibodies targeting immunostimulatory receptors such, as 4-1BB, DR5 and CD40, can stimulate anti-tumor immunity and provide therapeutic outcomes in multiple cancer types [195,196,197]. However, response rates using monoclonal antibodies are low and cancer often relapses, demonstrating the need for additional therapy. In models of renal and breast cancer combination treatment of α-GalCer with agonistic anti-DR5 and anti-41BB antibodies or agonistic anti-CD40 and anti-4-1BB antibodies led to a significant increase in tumor regression compared to monoclonal antibody therapy alone, with 80% of mice having complete tumor regression [120,121]. Importantly, the combination therapy lost its efficacy in Jα18^−/−^ iNKT cell mice, demonstrating that iNKT cells were essential in the combination therapy [121]. In mouse models of melanoma and breast cancer, combination treatment with α-GalCer and immunostimulatory cytokines IL-12 or IL-21 increased tumor regression and overall survival compared to monotherapy [122,123]. Both combination therapies increased NKT cell toxicity and were dependent on IFNγ production, demonstrating an increased anti-tumor immune response [122,123]. Taken together, a combination of iNKT cell immunotherapy with immunostimulatory cytokines or monoclonal antibodies increases anti-tumor immune function and tumor regression. However, there are concerns over the safety of immunostimulatory antibodies based on the toxicity of anti-CD40 agonistic antibodies in patient trials [198]and the disastrous results of the initial human phase 1 trial with an agonistic anti-CD28 antibody [199].

## 8. Next Steps and Challenges

The biggest challenge facing iNKT cell immunotherapy is the low infiltration and reduced function of iNKT cells in cancer patients [35,36,37,38]. Coupled with the downregulation of CD1d on tumor cells, treatment efficacy can be limited [50]. Furthermore, the induction of an anergic phenotype by iNKT cells following treatment with free α-GalCer, reduces the therapeutic benefit of multiple doses [200]. Another challenge is in obtaining large numbers of autologous DCs and iNKT cells from immunosuppressed cancer patients, limiting the number of cells that can be expanded and adoptively transferred [112,201]. Furthermore, it takes weeks to culture and differentiate cells for adoptive transfer, causing some patients to become ineligible for treatment or succumb to their disease [112]. To address these challenges, many modifications to iNKT cell immunotherapy have been examined or proposed.

### 8.1. Alternatative Glycolipid Delivery

Due to the low efficacy of free α-GalCer and the difficulty obtaining large quantities of autologous DCs, alternative methods to deliver α-GalCer are needed. Therefore, some studies have examined the potential for α-GalCer to be delivered through vectors such s, nanoparticles, artificial antigen-presenting cells, exosomes, and liposomes. Compared to free α-GalCer, delivery of vector bound α-GalCer increased iNKT cell expansion and cytokine release, resulting in decreased tumor burden [124,125,126,127,128,129]. This was largely due to the increased uptake and presentation by DCs and increased downstream NK and CD8 T cell responses [124,127]. Interestingly, treatment with vector-bound α-GalCer did not induce an anergic phenotype in iNKT cells [130,202]. This is potentially due to the reduction in B cells presenting α-GalCer to iNKT cells [60]. Vector-delivered α-GalCer is almost exclusively presented by DCs, which express costimulatory molecules that limit anergy induction in iNKT cells [61,124,127,200]. Interestingly, delivery of α-GalCer through exosomes, liposomes, or nanoparticles generated similar results, all increasing efficacy through similar mechanisms, demonstrating that the vector used can be used interchangeably [124,125,126]. An important, thing to note is that all studies examining vectored α-GalCer have been carried out using the B16 melanoma model. It remains to be seen whether α-GalCer delivered using vectors maintains its therapeutic benefit over free α-GalCer in tumors will lower immune-stimulating activity.

### 8.2. Alternatative Glycolipids

Almost all clinical trials examining iNKT cell immunotherapy have used α-GalCer (KRN7000) to stimulate iNKT cells; however, there is a growing list of modified glycolipids that may provide increased therapeutic benefit. Chemical modifications to α-GalCer have generated multiple analogs that produce a stronger T_h_1 cytokine response from iNKT cells. For example, α-C-GalCer replaces the O-glycoside linkage found in α-GalCer with a C-glycoside linkage resulting in increased IFNγ and IL-12 production [63,203,204]. Importantly IL-4 production from iNKT cells stimulated by α-C-GalCer was not increased, demonstrating increased skewing towards a T_h_1 phenotype [204,205]. While α-C-GalCer showed increased therapeutic benefit compared to α-GalCer preclinical trials in a metastatic melanoma model [203,206], α-C-GalCer does not stimulate human iNKT cells very well [207,208]. However, the T_h_1 skewing and therapeutic benefit of α-C-GalCer in preclinical models demonstrates proof of principle that iNKT stimulation via alternative glycolipids could produce benefits in human patients.

7DW8-5 has a shorter acyl chain which ends in a fluorinated benzene ring [209]. Compared to α-GalCer, 7DW8-5 has a stronger binding affinity to CD1d and the TCR of iNKT cells, leading to greater production of IFNγ and IL-2 [209]. While research examining 7DW8-5 therapeutic potential in cancer models is lacking, stimulation of iNKT cells with 7DW8-5 increases T_h_1 and CD8^+^ T cell responses in multiple vaccine models [209,210,211]. Importantly, unlike α-C-GalCer, 7DW8-5 strongly stimulates human iNKT cells in vitro [208,209]. Furthermore, in a malaria vaccine model, 7DW8-5 stimulated iNKT cells in rhesus macaques, whose iNKT cells are similar to humans, further demonstrating the potential for 7DW8-5 in human clinical studies [210,212,213]. Further examination in cancer models is needed.

Similarly, a modified glycolipid called ABX196 has generated promising results as a vaccine adjuvant. In both mice and humans, administration with ABX196 greatly increased iNKT production of IFNγ, but not IL-4, demonstrating increased T_h_1 skewing [214]. In melanoma, colon carcinoma, and bladder cancer models, ABX196 alone and in combination with anti-PD-1 increased tumor regression and overall survival [215]. ABX196 in combination with anti-PD-1 is currently being examined in a phase I clinical trial (NCT03897543).

Recently, a new glycolipid antigen commonly referred to as RK, has been synthesized [216]. RK-loaded-DCs increased IFNγ production from both human and mouse iNKT cells and promoted long-term T-cell memory [216]. Strikingly, mice treated with RK-loaded-DCs had complete elimination of melanoma metastasis, whereas mice treated with α-GalCer-loaded-DCs had the minimal therapeutic benefit, demonstrating increased therapeutic efficacy [216].

While the alternative glycolipids increase T_h_1 cytokine production and the anti-tumor function of iNKT cells, research is still limited. While direct comparisons with α-C-GalCer or RK show improved efficacy compared to α-GalCer without adverse effects, whether this holds true in humans is requires confirmation [206,216]. It remains to be seen whether any of the alternative glycolipids can improve the therapeutic benefit of iNKT cell cancer vaccines compared to α-GalCer. Furthermore, vectored alternative glycolipids may provide increased therapeutic benefit compared to vectored α-GalCer.

### 8.3. Induced Pluripotent Stem Cell-Derived iNKT Cells

Cancer patients often have reductions in the number and function of iNKT cells, limiting the efficacy of iNKT cell immunotherapy [35,36,37,38]. Furthermore, it can be difficult to obtain sufficient PBMCs from immunosuppressed cancer patients to expand and deliver iNKT cells ex vivo [112]. Therefore, a source of iNKT cells that can be delivered to supplement iNKT cells in cancer patients would be beneficial. One strategy would be to use induced pluripotent stem cells (iPSCs) derived from patient tissue [217]. iPSC-NKT cells have been generated via the nuclear transfer of stimulated splenic C57BL/6 NKT cells into embryonic stem cells and reprogrammed with retroviral vectors expressing Oct3/4, Sox2, Klf4, and Nanog to induce pluripotency [137]. iPSC-induced iNKT cells are capable of secreting large amounts of IFNγ [137]. Furthermore, iPSC-derived iNKT cells maintain their anti-tumor function when transferred into mice [137]. Recently, similar results have been observed using iPSC-derived human iNKT cells transferred into immunodeficient mice [136].

### 8.4. Inhibition of Type II NKT Cells

iNKT cells and type II NKT cells play opposing roles in the regulation of tumor immunity and cross-regulate one another [31,32,82,83]. Thus, targeting type II NKT cells may represent a therapeutic approach to alleviate tumor-mediated immunosuppression of iNKT cells and permit anti-tumor immunity. One method for targeting type II NKT cells may be through lipids that alter or block type II NKT cell responses. A recently described isoform of the type II NKT cell sulfatide antigen, C24:2, was demonstrated to significantly reduce the development of lung metastases [218]. Interestingly, other isoforms of sulfatide, including C24:1 and C24:0, increased the development of lung metastases in murine models of colon carcinoma [218]. Future exploration of altered NKT cell antigens should consider effects on type II NKT cell stimulation.

The TLR9 agonist and cancer vaccine adjuvant, CpG ODN, is known to upregulate IFNγ production, but not IL-4 in iNKT cells [219,220]. CpG ODN-activated iNKT cells were protective in the B16 melanoma model [219]. Interestingly, DCs sensitized to CpG induced IFNγ production, but not IL-4 and IL-13 from type II NKT cells. [221]. This increase in IFNγ production was partly inhibited when anti-CD1d antibodies were added to the culture, indicating CpG-induced IFNγ production was CD1d-dependent [222]. Intriguingly, when Ja18^−/−^ mice bearing B16 melanoma tumors were treated with CpG, tumor growth was significantly reduced compared to CD1d^−/−^ mice (deficient in type I and type II NKT cells) [222]. CpG-treated Ja18^−/−^ mice had significantly higher proportions of IFNγ producing CD8^+^ T cells when compared to CD1d^−/−^ mice [219]. Moreover, wild-type mice exhibited the greatest reduction in tumor growth, suggesting that both NKT cell subsets could be influenced by CpG and contribute to potent antitumor activity [219]. However, conflicting studies suggest that CpG suppresses iNKT cell function and CpG-dependent B16 melanoma tumor growth inhibition could only be observed in the absence of type II NKT cells [223,224]. Thus, further investigation into the anti-tumor effects of CpG on both subtypes of NKT cells is required. Taken together, CpG and other T_h_1-polarizing adjuvants should be considered not only as iNKT cancer immunotherapy adjuvants, but also as a Th1-polarizing agent for type II NKT cells that may help to alter their immunoregulatory functions.

### 8.5. CAR-NKT Cells: Lessons from CAR-T Cells

In recent years, many improvements have been made to CAR-T cell therapy to increase efficacy, manufacturing, and safety [225]. Many of these improvements have seen both preclinical and clinical success that may be able to increase the efficacy of CAR-NKT cells as well.

Cancers are often heterogenic, expressing different markers in different regions of the tumor [226]. Therefore, treatment with CAR-T or CAR-NKT cells targeting a single antigen may miss cancer cells not expressing the targeted antigen, reducing efficacy, and leading to potential tumor relapse. Therefore, multiple strategies have been developed to overcome this. The development of bispecific CAR-T cells that can target multiple antigens has increased efficacy in both preclinical and clinical settings [227,228,229]. The conserved recognition of CD1d by NKT cells already provides this dual targeting specificity. Another option is the use of universal CAR-T cells, that use adaptors to target different antigens [230]. Universal CAR-T cells split their T cell signaling unit and antigen targeting domain leaving the CAR-T cell inert until the adaptor protein expressing the antigen targeting domain is added [231]. This may be especially useful for CAR-NKT cells due to their reduced risk of GvHD and CRS, allowing for use of allogenic donors [150]. This opens the option for premade universal CAR-NKT cells and adaptors ready to treat patients, overcoming the lengthy manufacturing process of generating cells from individual patients and allowing more people to receive treatment [232]. Furthermore, many new technologies have been developed to decrease the manufacturing time of CAR-T cells [233,234]. Newer technologies avoid the use of lentiviral delivery, avoiding the risk of loss or gain of function mutations in the CAR-T cells [235]. The newer technologies could be used to produce CAR-NKT cells faster and at reduced cost, leading to a more accessible treatment.

## 9. Concluding Remarks

iNKT cells play an important role in immunosurveillance and anti-tumor immunity [15,16,17]. Although preclinical studies have revealed effective approaches, many initial treatment strategies have generated little clinical success. Newer treatment strategies, including vectored α-GalCer, alternative glycolipids, and combination therapies, are showing improved preclinical results and highlight the need for clinical trials using these strategies. Furthermore, initial clinical results from the anti-CD19 CAR-NKT and anti-GD2 CAR-NKT clinical trials are promising, demonstrating that iNKT cell immunotherapy can be a platform to provide curative outcomes. 

## Figures and Tables

**Table 1:** Preclinical and clinical studies utilizing various iNKT cell immunotherapy strategies.

Strategy	Therapy Regimens	Preclinical Models or Clinical Trials	Outcome	References
**Free** **glycolipids**				
	α-GalCer (i.v.)	Multiple cancer models	Activation of iNKT, NK, T cells, increased IFN-γ and IL-12	[97,98]
	α-GalCer (i.p.)	Multiple cancer models	NK and iNKT mediated tumor cell elimination, elevated IFN-γ	[99,100]
**Adoptive** **transfers**				
	Tumor cells irradiated and loaded with α-GalCer	A20 lymphoma, Meth A sarcoma, VK*Myc model, Eµ-myc model	iNKT cells and effector T cells promote anti-tumor immunity, elevated IFN-γ and IL-12	[101]
	α-GalCer loaded dendritic cells	Multiple cancer models	Activation of iNKT cells	[51,52,102]
	α-GalCer loaded -APCs	Myeloma, NSCLC, and Head and neck cancer	Increased iNKT expansion and IFNγ production leading to stable disease	[103,104,105]
	Ex-vivo expanded iNKT cells	Multiple cancer models	Increased iNKT cytotoxicity, tumor regression and overall survival. Was model dependent	[17,52,106,107]
**CAR-NKT cells**				
	GD2 CAR NKT	Melanoma and neuroblastoma models	Cytotoxicity against GD2 positive tumors, increased Th1 cytokines and localization to tumor site	[108,109,110]
	CD62L^+^ CD19 CAR NKT cells	B cell lymphoma model	Prolonged persistence in vivo	[96,111]
	CSPG4 CAR NKT cells	Melanoma	Increased iNKT pro-inflammatory cytokine production	[112]
**CD1d-antibody fusion proteins**				
	Anti-HER2	Melanoma and Colon carcinoma	Increased iNKT pro-inflammatory cytokine production and cytotoxicity. Increase DC, NK, and CD8 T cells recruitment Increased tumor regression. Limited off-target effects.	[113,114]
	Anti-CEA	Colon carcinoma	Increased iNKT pro-inflammatory cytokine production and cytotoxicity. Increased tumor regression.	[113]
	Anti-CD19	Melanoma and Colon carcinoma	Increased iNKT pro-inflammatory cytokine production and cytotoxicity. Increased tumor regression.	[115]
**Combination therapies**				
	α-GalCer-loaded DCs + expanded iNKT cells	Head and neck cancer	Increased circulating iNKT cell number and IFNγ production.	[17,116]
	α-GalCer -loaded DCs + anti-PD-1 or anti-PD-L1 mAbs	Melanoma metastasis, Colon cancer, Hepatoma model	Prevented iNKT cell anergy and enhanced anti-tumor function overcomes CD8 T cell exhaustion in PD-1 resistant tumors	[117,118,119]
	α-GalCer + anti-4-1BB, anti-CD40, or anti-DR5	Renal and breast cancer models	Stimulate robust anti-tumor immunity	[120,121]
	α-GalCer + IL-12 or IL-21	Melanoma and breast cancer models	Increased tumor regression and overall survival, increased iNKT cell cytotoxicity	[122,123]
	Vector bound α-GalCer	B16 melanoma models	Increased iNKT cell expansion and cytokine release, prevented NKT cell anergy	[124,125,126,127,128,129,130]
	α-GalCer + Cisplatin	Mesothelioma Model	Increased pro-inflammatory cytokine gene expression and tumor regression	[131]
	α-GalCer + 5-FU	Colon cancer model	Increased NK cell cytotoxicity and coreceptor expression	[132]
	α-GalCer loaded APCs and lenalidomide	Multiple myeloma patients	Decreased cancer cell proliferation, angiogenesis. Increased T cell, NK cell and iNKT cell activation and expansion. Elevated iNKT cell cytokine production. Well tolerated in patients.	[133,134]
	α-GalCer loaded APCs and anti-DEC205 nanoparticles	B16-F10 melanoma model	iNKT cell-mediated activation of NK cells, DCs, and γδ T cells	[92]
	α-GalCer-loaded APCs and oncolytic VSV	4T1 breast cancer and ID8 ovarian cancer models	Induced immunogenic cell death and lead to increased overall survival	[134,135]
	α-GalCer + iPSC-iNKT cells	EL4 T cell lymphoma	Enhanced anti-tumor activity and tumor regression	[136,137]

i.v., intravenous; i.p., intraperitoneal.
